# Changes in Food Environment Patterns in the Metropolitan Area of the Valley of Mexico, 2010–2020

**DOI:** 10.3390/ijerph19158960

**Published:** 2022-07-23

**Authors:** Ana Luisa Reyes-Puente, Dalia Guadalupe Peña-Portilla, Sofía Alcalá-Reyes, Laura Rodríguez-Bustos, Juan Manuel Núñez

**Affiliations:** 1 Environmental Sustainability, Universidad Iberoamericana, Mexico City 01219, Mexico; a2180824@correo.uia.mx (A.L.R.-P.); a2169877@correo.uia.mx (S.A.-R.); 2 Facultad de Ingeniería, Universidad Nacional Autónoma de México, Mexico City 04510, Mexico; daliapp@comunidad.unam.mx; 3 Instituto de Ecología, Universidad Nacional Autónoma de México, Mexico City 04510, Mexico; 4 Centro Transdisciplinar Universitario para la Sustentabilidad, Universidad Iberoamericana, Mexico City 01219, Mexico

**Keywords:** food environments, food deserts, food oases, food swamps, sustainable food system

## Abstract

The concept of food environment refers to the opportunities; environments; and physical, economic, political, and socio-cultural conditions that frame the interaction of people with the food system and shape decisions about food acquisition and consumption. This study analyzes the relationships between the characteristics of urban environments and the availability of retail food through the evaluation of physical and financial access to food in the Metropolitan Area of the Valley of Mexico (MAVM) between 2010 and 2020. Using Geographic Information Systems (GISs), both physical access through network distance to economic food retail units and financial access through socioeconomic status at the block scale were evaluated. The network distance and socioeconomic status results were used as criteria for the spatially explicit classification of the MAVM into food deserts, oases, and swamps. Food deserts are the most abundant food environments but only increased in the third and fourth metropolitan contours. Swamps have increased throughout the city, related to the proliferation of convenience stores that have replaced grocery stores. This study contributes evidence at a local and regional scale required for the future urban planning of the MAVM and for public health and sustainability programs focusing on treating food-related diseases.

## 1. Introduction

Approximately 55% of the world population lives in cities whose geographical environments must satisfy food requirements and encourage the choice of healthy, sustainable foods that will guarantee food security and nutrition for all their inhabitants [1]. To address this global problem, the food system proposal has emerged as a concept for understanding the environment, people, inputs and their processes, institutions, and infrastructure in relation to food production, processing, distribution, preparation, and consumption activities as well as the socioeconomic and environmental consequences of the latter [2]. Food systems have three components: food supply chains, food environments, and consumer behavior [3].

Of these components, the food environment is crucial because it refers to the socio-cultural, political, and economic environments that determine the interaction of people with the food system and shape the context of food acquisition and consumption [4,5]. This includes the availability of food products and consumer preferences. The food environment is, therefore the territorial expression of food systems that makes it possible to promote sustainable diets and implement interventions to eradicate diseases associated with food insecurity, such as obesity, diabetes, hypertension, and malnutrition [5,6].

Some factors considered in shaping food environments are retail units, the characteristics, infrastructure of access to stores, and the personal determinants of consumer food choices. These include the physical (proximity) and financial (affordability) accessibility of food, promotion and advertising, and the quality and safety of the latter [3].

Physical access to food depends primarily on the built environment, such as the presence of food outlets and adequate infrastructure for access [3]. This factor has been calculated through various measures commonly used to measure accessibility, such as the number of commercial establishments in a given area, the number of commercial establishments within a sphere of influence, and the minimum distance between commercial establishments and households [7].

The spatial distribution of retail stores is related to the type of commerce involved. Commercial premises that belong to a traditional retail channel are small premises or markets [8], while premises that belong to a modern retail channel are convenience stores or supermarkets [9]. Regarding measuring the accessibility of commercial establishments, the analysis of walks using Geographic Information Systems (GISs) estimates reality because it shows the easiest way to access retail stores and the distance of the routes a person must take [10,11,12,13,14]. 

Although food environments vary spatially, lower-income households have been found to spend more of their total capital on food [15,16]. Whereas high-income households invest more economic resources, this represents a smaller portion of the total budget, which is usually associated with healthier diets [17,18]. Generally, countries in the global south spend approximately 50% of their income on unprepared food, whereas countries in the global north spend less than 10% on this [19]. Based on 2020 data, the United States reported that lower-income households spent approximately USD 4099 on food (27% of their income), whereas higher-income households spent an average of USD 12,245 (7% of their income) [20].

Several studies have focused on developing analysis frameworks in food environments and have established three categories of access to food for urban food environments [21,22,23]. The first category is food deserts, defined as a deprived areas where residents have barriers to accessing nutritious, affordable food [10]. Conversely, the second category is food oases, defined as a privileged area where residents have access to healthy food [24]. Finally, the third category is food swamps, defined as an area where residents have access to copious amounts of food with a high caloric content, where the supply inundates healthy food options [4,10,25].

Food environments have been studied extensively in the context of the Global North [4,10,11,26]. As the term became more complex and its political use increased, it began to be studied through its empirical application in food environments in the Global South, in countries in Africa, Asia, and Latin America [27,28,29,30,31].

There is a dearth of literature and research on food environments in Mexico. They can be summarized as studies that address the concepts of food deserts and oases from a regional perspective [14,32], calculations of access rates to food [13,33], and cartographic representations of food deserts [32]. Researching food environments in Mexico is urgently required due to the prevalence of diet-related chronic diseases in the country, particularly diabetes, obesity, and nutrient deficiency [34].

Nutrition is the driving force behind the changes required to achieve a sustainable future. Access to safe, sufficient, nutritious food is a human right, and policies that promote agriculture and food systems that operate based on the nutritional quality of food are required [34]. This research contributes to the applied research on food environments through a methodological proposal for estimating urban food environments in the Metropolitan Area of the Valley of Mexico (MAVM) in 2010 and 2020. The theoretical foundation required rethinking concepts to be appropriate to the context of the study area. Therefore, a geographical analysis approach is adopted to recognize the level of financial and physical access to food. In this way, this study establishes associations between affordability and proximity of people to quality food.

## 2. Materials and Methods

### 2.1. Study Area

**Figure 1:**
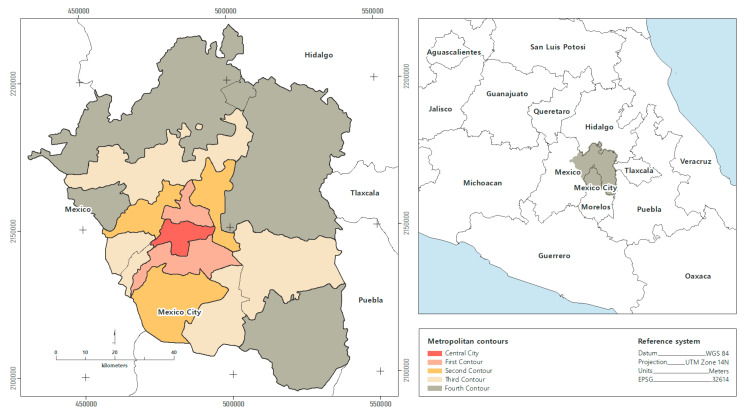
Study area.

The Metropolitan Area of the Valley of Mexico (MAVM) comprises an area of 7854 square kilometers, encompassing the 16 boroughs in Mexico City (CDMX) and 60 conurbation municipalities in the State of Mexico and Hidalgo. A total of 21.8 million people live in the MAVM, the most important economic region in the country due to the provision of services, and the region is characterized by a rapid urbanization process [35,36]. The territorial delimitation of the study area in this research takes up the proposal of [37] that divided the MAVM into metropolitan contours (Figure 1). These contours show the conurbation of Mexico City with the State of Mexico and explain the process of disaggregation of the population from the central city to more peripheral contours.

### 2.2. Methodology

The methodology used in this study focused on recognizing two aspects of food environments: physical (accessibility) and financial (affordability) access to food. A proximity analysis was conducted to assess physical access based on the network distance involved in walking to retail food units from households based on the centroid of urban blocks in the MAVM. For financial access, the socioeconomic status of people at the urban block scale was estimated from three different socioeconomic indices to obtain, through a principal components analysis, a single score to arrange the urban blocks in the MAVM according to the predominant socioeconomic status. Based on the integration of accessibility and affordability, blocks in the MAVM were classified as deserts, oases, and food swamps. The methodology was used for 2010 and 2020 to compare food environment patterns in the same geographical area.

#### 2.2.1. Physical Access to Food: Network Distance Analysis to Retail Food Units

Retail trade units (RTUs), food sources, were classified into supermarkets, convenience stores, and local commerce. The typology was obtained from the International Industrial Classification of the North American Industrial Classification System [38], whose georeferenced data are available in the National Statistical Directory of Economic Units (Spanish Acronym DENUE) (Table 1).

To calculate the distance from people to food, we used the network distance between commercial establishments and households, calculated on the basis of each of the centroids of the blocks with population in the MAVM to all the retail food units in the city’s road network. This was calculated using the QNEAT3 tool (QGIS Network Analysis Toolbox 3), which makes it possible to construct a network distance matrix between intersection points through the OD-Matrix from Points (n:m) geoprocess for various means of transport, such as walking, wheelchairs, bicycles, and various motorized forms of transport [46]. This action produced a table with n × m records of the distance between the block centers and the retail food units through the road network for each of the selected means of transport.

#### 2.2.2. Financial Access to Food: An Estimate of Socioeconomic Status at an Urban Block Scale

In the conceptualization of this study, socioeconomic status (SES) was chosen as a suitable indicator to calculate people’s financial access to food. From a marketing perspective, SES has been used by academics as the basis for income segmentation [47]. Moreover, researchers in the field of health devote a great deal of attention to the concept of consumer SES as a determining aspect of people’s health and nutritional status [48]. In fact, people with a high SES are more likely to consume healthy, nutritious food, whereas people with a low SES are more likely to consume foods with low nutritional quality [49,50]. Several authors have suggested that access to healthy food varies by socioeconomic status and type of food store [49,50,51].

The calculation used the approach proposed by the Mexican Association of Market Intelligence and Public Opinion Agencies (Spanish acronym AMAI), which measures the satisfaction of the needs of all household members [52]. SES represents the ability to access a set of goods andlifestylese that enables consumers to be segmented based on the mediation of demographic, social, technological, and media factors, which, through a complex social dynamic, lead to a determined lifestyle [53]. The “NSE 2010 Rule” combines six numerical and categorical variables: educational attainment of the head of household, number of bedrooms, number of full bathrooms, number of employed persons aged 14 years and over, number of cars, SUVs, and vans, and having fixed Internet in the home [52].

To estimate the predominant SES at the urban block level, a methodology adapted from the multivariate analysis was used [54]. This involves conducting a Principal Component Analysis (PCA) based on three different socioeconomic indices to obtain a single score that enables urban blocks to be arranged according to the predominant socioeconomic status Subsequently, all the blocks are arranged in descending order from the first component, and seven cut-off points are established for each SES by applying a cumulative distribution to the proportion of the cumulative population of the blocks, known as the AMAI 8 X 7 rule [53]. The data used are drawn from the 2010 and 2020 Population and Housing Censuses undertaken by the National Institute of Statistics and Geography (INEGI).

The socioeconomic indices constructed from the available data are shown below:

Durable Goods Index ($I_{B}$)

$$I_{B}=\frac{\mathrm{Number}\;\mathrm{of}\;\mathrm{dwellings}\;\mathrm{with}\;\mathrm{computers}\;\mathrm{in}\;\mathrm{the}\;\mathrm{AGEB}\;\left(\mathrm{basic}\;\mathrm{geostatistical}\;\mathrm{area}\right)}{\mathrm{Dwellings}\;\mathrm{with}\;\mathrm{some}\;\mathrm{of}\;\mathrm{these}\;\mathrm{goods}\;\left(\mathrm{computer},\;\mathrm{refrigerator},\;\mathrm{washing}\;\mathrm{machine},\;\mathrm{television}\right)\;}$$

Educational Attainment Index ($I_{E}$)

$$I_{E}=\frac{\mathrm{Average}\;\mathrm{years}\;\mathrm{studied}\;\mathrm{in}\;\mathrm{the}\;\mathrm{AGEB}}{\mathrm{Maximum}\;\mathrm{average}\;\mathrm{years}\;\mathrm{studies}\;\mathrm{in}\;\mathrm{all}\;\mathrm{the}\;\mathrm{AGEBs}\;}$$

Higher Education Index ($I_{\mathrm{ES}}$) (the following variables were considered to calculate the Higher Education Index in 2010: percentage of the population aged 25 years and over with at least one approved degree in higher education/population aged 25 years and over. Due to the fact that, in 2020, the same variables were not found as those in 2010, the most similar ones were used: population aged 18 years and over with post-basic education/population aged years 18 and over.)

$$I_{\mathrm{ES}}=\frac{\mathrm{Number}\;\mathrm{of}\;\mathrm{individuals}\;\mathrm{over}\;25\;\mathrm{and}\;\mathrm{with}\;\mathrm{higher}\;\mathrm{education}\;\mathrm{in}\;\mathrm{the}\;\mathrm{AGEB}\;}{\mathrm{Number}\;\mathrm{of}\;\mathrm{individuals}\;\mathrm{over}\;25}$$

The PCA was performed using the Python programming language from the construction of a matrix of correlation coefficients and the results focused on recognizing the importance of each variable in the distribution of data and coordinates. Its applicability was evaluated using Bartlett’s sphericity test and the Kaiser–Meyer–Olkin (KMO) adequacy test to verify the independence of each variable [55] and the adequacy of the sample [56]. A KMO value close to 1.0 indicates that the data are suitable for the PCA [57].

#### 2.2.3. Criteria for the Definition of Urban Food Environments: Deserts, Oases, and Swamps

Based on the results of the analysis of physical and economic access, we adjusted the proposal of [13,14] to classify the food environments in each block of the MAVM into food oases, swamps, or deserts. Food deserts are defined as spaces in which the inhabitants lack access to healthy food due to physical and economic barriers [7,14]. In this study, food deserts are blocks with a low SES and a radial distance to supermarkets of more than one km. This distance includes turns, crossings, and waiting times at intersections with an approximate walking time of 20 min at an average urban speed of three km per hour [58]. More than one km is considered an insufficient distance to cover food accessibility [13,14].

Conversely, food oases are commonly defined due to the proximity of homes to supermarkets [6]. In this study, food oases are blocks with a high SES and a radial distance to supermarkets, convenience stores, and local businesses of less than 0.5 km. This distance includes turns and crossings and represents an approximate, adequate time of 10 min of actual walking [59]. Finally, food swamps are blocks with any SES and a shorter radial distance to convenience stores than any other type of business.

The main characteristic of food swamps is the proximity to outlets with an excess of food associated with a poor diet [25,60]. This type of food is ultra-processed with artificial colors and high sugar, saturated fat, and sodium contents and low protein, fiber, and vitamin contents [61].

Supermarkets are large commercial establishments characterized by their wide range of fresh and packaged foods [39]. Convenience stores offer foods with less nutritional value, such as sugary and alcoholic beverages, snacks, canned foods, and other processed and ultra-processed foods. Grocery stores include greengrocers, butchers, poultry stores, and fishmongers [62]. The latter has a broader range of food than convenience stores but less variety than supermarkets. 

## 3. Results

### 3.1. Network Distance to Economic Units of Food Retail Trade

**Figure 2:**
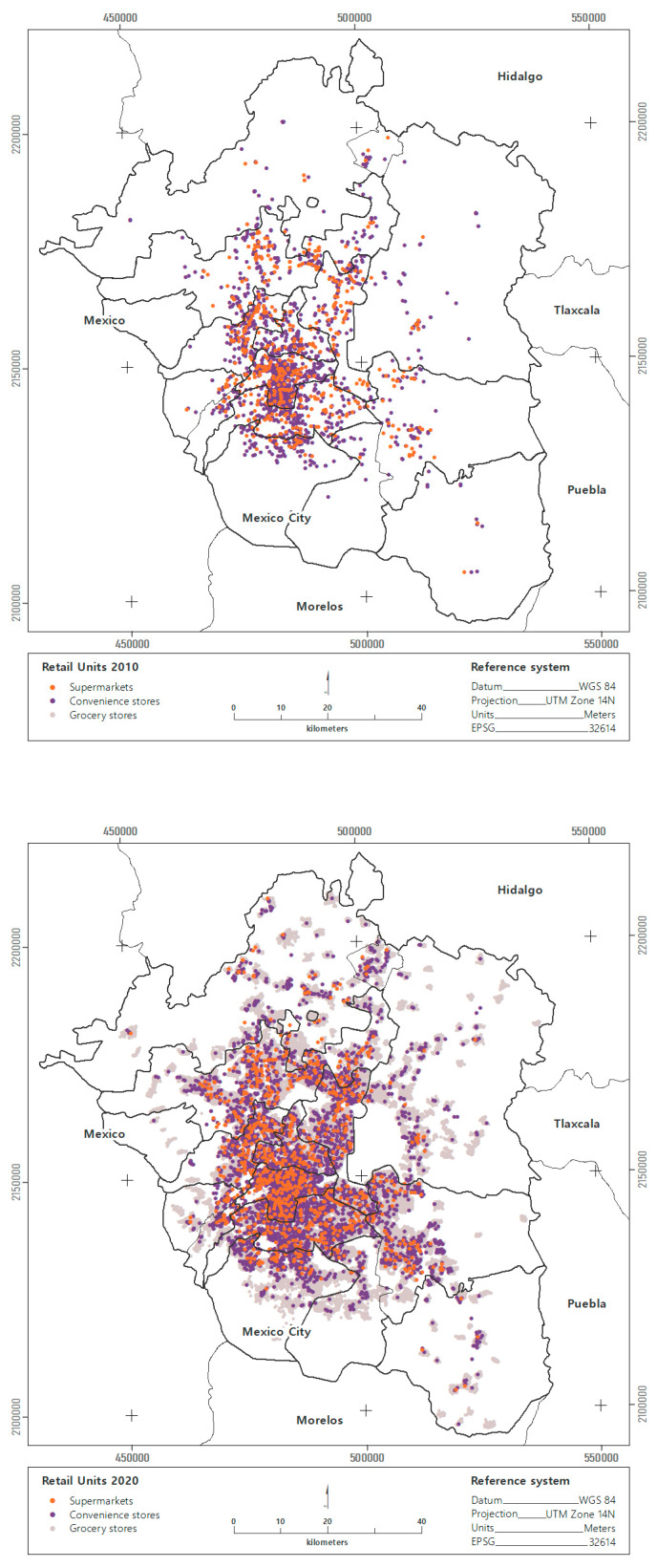
Food retail units in the MAVM, 2010–2020.

The average distance to retail food units (FRUs) has changed during the past ten years in the MAVM. The most significant change was recorded for convenience stores, for which the average distance decreased by 50% between 2010 and 2020, from 927 to 470 m. The average distance to supermarkets slightly decreases by 20% in the same period from 1488 to 1188 m. The distance to grocery stores remained unchanged. In both years, one grocery store was reported at a distance of 90 m, corresponding to one local retail store per block in the MAVM (Figure 2).

Although grocery stores are the most common FRUs in the MAVM, they are the retail units that grew the least between 2010 and 2020, increasing by 6%. This type of FRU went from 156,617 to 165,772 establishments. The second most common type of FRU is convenience stores, which are the FRUs that grew the most between 2010 and 2020, tripling in number. Where in 2010, there were only 1697 convenience stores. By 2020, this number had increased to 5618, meaning that, for the past ten years, one convenience store has opened every day. Supermarkets are the least common FRU, and their growth in the MAVM was 49% for the same period. By 2010, there were 550 supermarkets, and by 2020 this type of FRU had increased to 1069 (Figure 2).

The urban contours of the MAVM have shown significant patterns of change in the growth and distribution of supermarkets and convenience stores. The total number of retail units in the central city fell by 12% between 2010 and 2020. However, this change results from the 17% decrease in grocery stores. The number of supermarkets increased by 50%, while convenience stores doubled. In the first contour, the total FRUs decreased by 4% between 2010 and 2020. There are 7% fewer grocery stores, and the most significant change occurred with convenience stores, which tripled in ten years. In the second, third, and fourth contours, the total number of retail trade units increased between 2010 and 2020.

The main pattern of change was observed in convenience stores, which tripled in the second contour, quadrupled in the third contour, and quintupled in the fourth contour of the MAVM between 2010 and 2020. Thus, for example, in areas with a low SES, located in the last two metropolitan contours, for every supermarket and convenience store that opened, 19 grocery stores closed in the past ten years.

### 3.2. Socioeconomic Status at the Urban Block Scale

The first dimension of the PCA constructed from the three socioeconomic indices explained 91.8% of the total variability of the data in 2010 and 88.3% in 2020. The KMO test obtained a value of 0.74907 and Bartlett’s test of sphericity χ^2^ = 387,612.9 (*p* = 0.00) for the 2010 data. In 2020, KMO obtained a value of 0.71003 and Bartlett’s test of sphericity χ^2^ = 454,542.1 (*p* = 0.00). This score was highly acceptable, indicating adequate PCA results for both dates.

According to the most recent methodological note from [52], the highest SES (A/B) spends an average of 28% of their total income on food and invests 10% in education. At this level, 82% of households have a householder with professional or postgraduate studies and 98% have fixed Internet. The average SES (C+, C, C−) spends between 32 and 38% on food, 24% on transport and communication, or owns at least one transport vehicle. At this level, 75% of households have a householder with an academic level above primary school, and between 52% and 93% of households have fixed Internet. The low SES (D+, D, and E) allocates between 42–52% of its expenditure to food, between 11 and 16% to transportation and communication, and only 7% to education. At this level, 75% of households have a householder with an academic level above primary school, and between 52% and 93% of households have fixed Internet.

The population in the MAVM increased from 20.1 to 21.8 million between 2010 and 2020, reflecting an annual growth rate of 0.81%, concentrated in contours 3 and 4. The total population with a high SES (A/B) decreased 1% between 2010 and 2020 in the MAVM. In the middle SES (C+, C, C−), the total population increased by 34%; in the low SES (D+, D, E), it decreased by 4%. 

**Figure 3:**
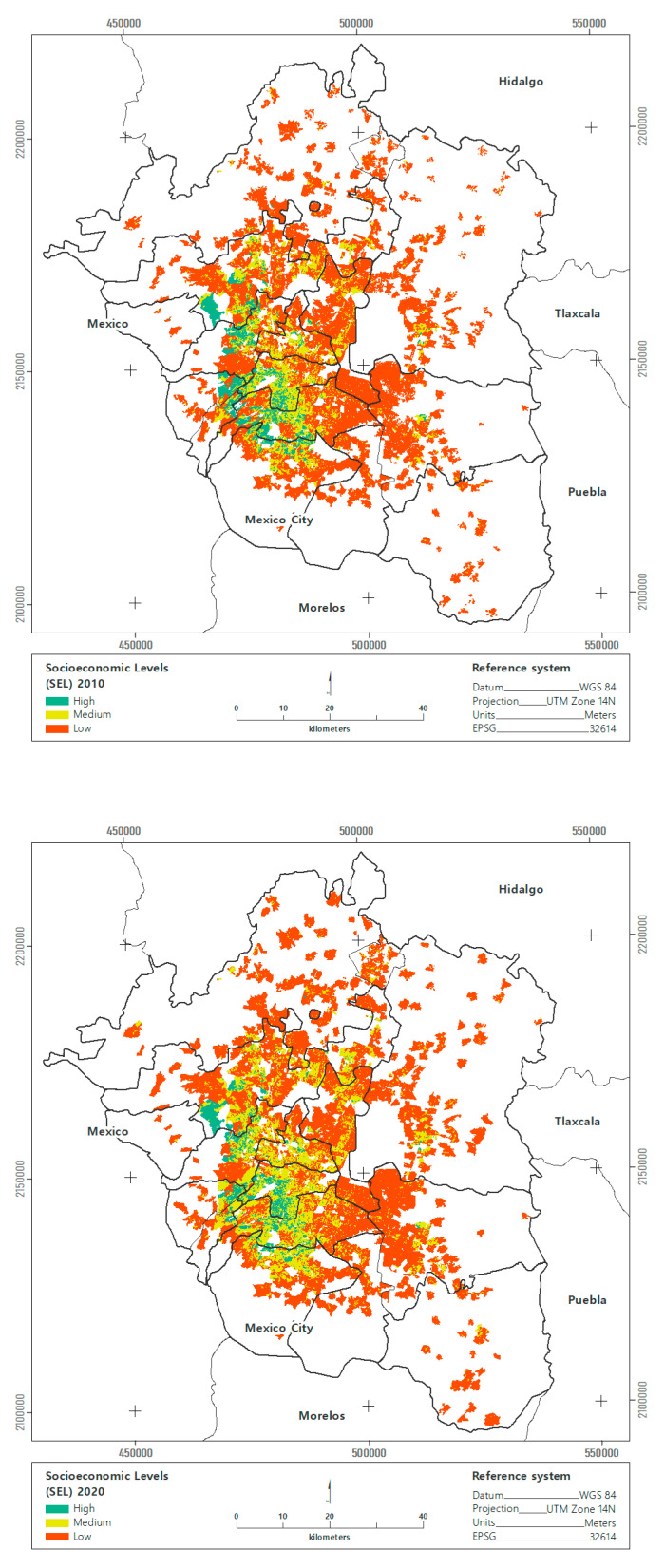
Socioeconomic status for the MAVM, 2010–2020.

SES distribution differs between the urban contours of the MAVM. In the Central City, the greatest change was recorded in the 32% decrease in blocks with low SES. Blocks with a high SES increased by 23% and blocks with a medium SES increased by 9% for the period during 2010–2020. In the first contour, blocks with a high SES decreased by 10%, in the medium SES they increased by 37%, and in the low SES they decreased by 8% between 2010 and 2020. In the second contour, blocks with high SES decreased by 10%; those with medium SES increased by 39% and those with low SES increased by 4%. In the third contour, blocks with high SES increased by 7%. The most significant change was the 77% increase in blocks with medium SESs, while those with low SESs increased by 39%. In the fourth contour, the number of blocks with high, medium, and low SESs doubled between 2010 and 2020 (Figure 3).

### 3.3. Urban Food Environments

Food deserts were the most abundant environment in the MAVM in 2010 and 2020, a period when blocks classified as deserts decreased by 4.5%. Oases were the second most common environment and showed a changing trend where the number of blocks tripled during 2010–2020. Swamps were the least common environments, but their change in trend is the most notable because the number of blocks tripled during the same period (Table 2).

Food deserts concentrated the largest number of inhabitants in both 2010 and 2020. The change in trend indicated that the population in food desert conditions decreased by 37% for the same period. The lowest number of inhabitants was concentrated in food oases, and between 2010 and 2020, the population in food oases increased by 30%.

Food swamps recorded the most significant pattern of change because the population doubled between 2010 and 2020. The difference between the population in deserts compared to oases showed that in 2010, there were 40 times more people in deserts than oases. In 2020, the proportion decreased to 18. The population difference between deserts and swamps showed that in 2010 there were 19 times more people in deserts than swamps. By 2020, this proportion had decreased to five.

**Figure 4:**
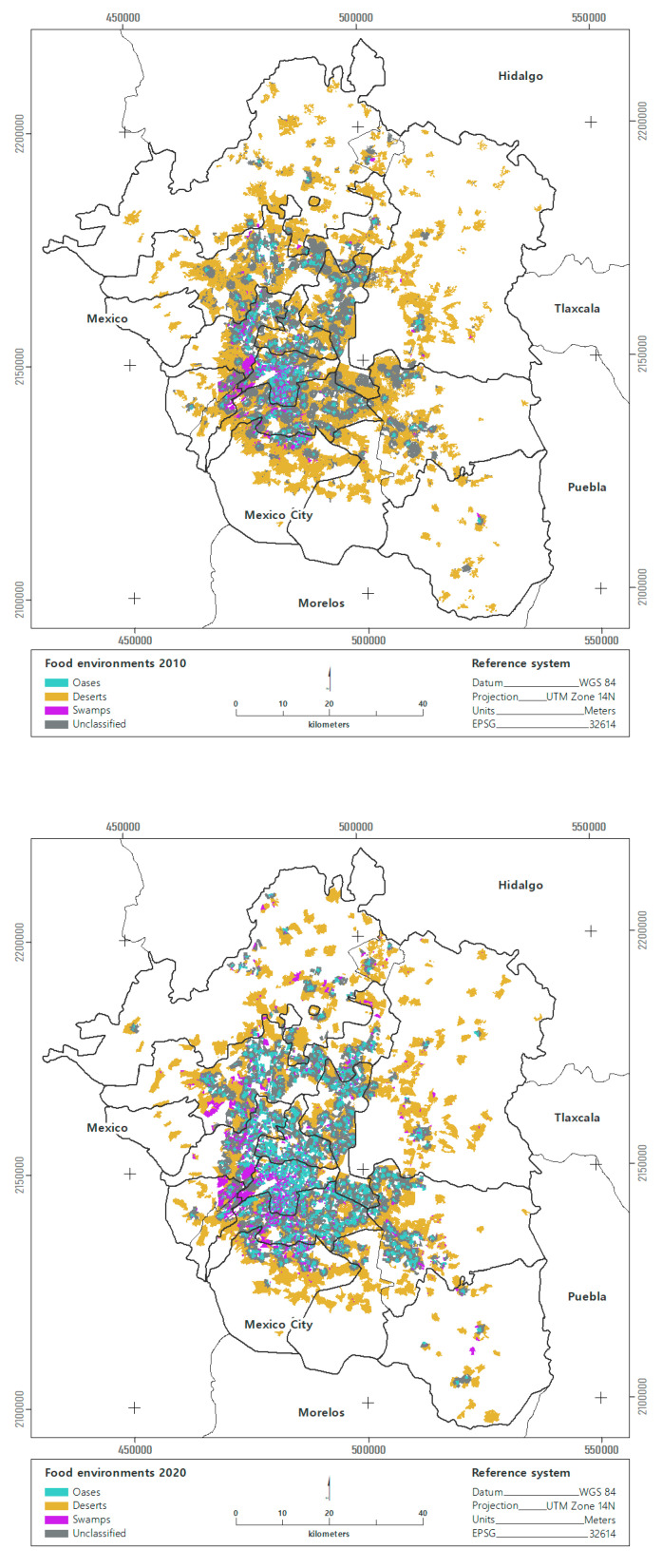
Changes in urban food environments of the MAVM, 2010–2020.

Food environments showed a differential distribution in the MAVM contours. For the period during 2010–2020, oases, deserts, and swamps were distributed among the Central City and the first and second contours. In the third and fourth contours, only deserts and swamps were recorded. The most important pattern of change in all the metropolitan contours is the increase in the number of swamps between 2010 and 2020. Most oases were concentrated in the Central City, where the most significant pattern of change was registered between 2010 and 2020 as a result of the decrease in food deserts and the increase in swamps (Figure 4).

## 4. Discussion

Food environments are created through complex social and urban historical processes over prolonged periods of time that shape food access, preference, and consumption at the individual level [22]. In the case of the MAVM, rapid population growth and disorganized urban growth exacerbated the problems associated with food insecurity in certain metropolitan areas. For example, food deserts were the most common environment in the period during 2010–2020 throughout the city, but were concentrated, together with swamps, in the peripheral zones of the third and fourth contours. The food preferences of the population, together with the needs for physical and financial access, therefore, determine the way to access healthier foods.

The population living in a food desert is at higher risk of suffering from diseases associated with malnutrition [63,64]. Accordingly, the research on food environments has focused on recognizing deserts as the sites with the greatest exposure to health problems. This research adopted a different perspective because, in the MAVM, food swamps reflect a more severe problem, since they constitute an environment without physical access to healthier food or the ability to pay for it. This type of environment is regarded by [65] as obesogenic since the population within them has a greater risk of suffering from the double burden of malnutrition with obesity and undernutrition.

The results of this study show that one of the driving factors in the increase in food swamps is the proliferation of convenience stores replacing grocery stores. This process of change is relevant because convenience stores are the economic unit with the lowest stock of healthy foods, offering foods with a high caloric content, compared to supermarkets and grocery stores that provide healthier, more diverse foods. In this respect, a more in-depth study of this food environment in the MAVM could become a central axis of social programs to attempt to reverse the spread of obesity among the poorest urban populations.

The international literature recognizes that the excessive supply of foods with a high caloric and low nutritional content has displaced the supply of healthy foods [4,10,25]. The intersection between financial limits, or the inequality of opportunities to acquire food, even if it is present, and limited physical access, which occurs when there are no healthy options for the population, encourage the proliferation of food swamps. In this study, we considered that swamps are the least favorable food environment for food security because, in these places, there is no freedom of decision for the population regarding the type of food they wish to consume. For example, in the MAVM, there are food swamps in all geographic contours and at all socioeconomic levels. However, food swamps are concentrated in the third and fourth contours, where people have lower SESs and, in addition, are conditioned to having access to low-quality food.

These results highlight a set of social shortcomings that can be addressed from a public health perspective. Ref. [34] urges all governments to meet the food needs of the vulnerable population and to encourage social protection programs for the population most exposed to diseases associated with malnutrition, such as obesity, diabetes, and hypertension. In the third and fourth contours of the MAVM, there is an urgent need to increase the supply of healthier foods because the pattern of change shows a 37% increase in the average SES. Accordingly, even if the population increases its purchasing power in the coming years, it will not be guaranteed physical access to healthier foods.

In this respect, the objective of food security on international agendas is to achieve a nutritious, balanced, accessible, and affordable diet for all inhabitants [34]. To this end, the care strategies for each metropolitan area will therefore have to be differentiated and at the same time integrated. For example, a food swamp located in the first or second contour of the MAVM where the population has a medium or high SES can be resolved through access to retail food units with organic products from local producers. In contrast, a food swamp located in the third and fourth contours characterized by low SESs could consider designing strategies to access healthier foods given their proximity to rural productive units.

The research based on the analysis of food environments is in its infancy [4]. Despite this, the contributions of research on food environments are increasingly important to understanding the effect of the availability of a certain type of food on the individual choices a person makes to feed themselves [66]. People exposed to food environments with little nutritional value perceive junk foods as normal and want to consume them regardless of their physical or financial availability [33]. Any political action to address the marketing of unhealthy products should guide people’s choices by making healthy foods available, while also respecting their preferences and social norms regarding consumption.

In any public policy scheme, solving the problem of physical access to healthy food is a priority because it has been established that the environment shapes decisions [4,33]. Once the environment has been modified, norms and social preferences regarding consumption respond. Ref. [67] found that moving to a neighborhood with a higher percentage of healthy foods is associated with an increased intake of healthy foods, especially fruits and vegetables, in people who previously did not consume them.

The results of this study provide evidence at a local and regional scale that, regardless of the SES of the population, there are urban contours of the MAVM where the population has less access to healthy foods. This type of information is essential for the future sustainable urban planning of the MAVM and for public health programs designed to treat diseases associated with poor nutrition. For example, the political agenda of the Mexican government is working on the National Strategy of Healthy, Fair, and Sustainable Food whose main objective is to contribute to the progressive realization of the right to adequate food [68]. The information presented in this study contributes to this policy program at the regional level because it identifies sites, at the city block scale, where it is essential to balance the supply of available food with healthy food. Additionally, it highlights the spheres of action where consumption norms and preferences can be satisfied because the food supply is balanced, and the purchasing power exists to promote healthy diets.

Regarding the sustainability agenda, there is a need to focus on creating sustainable food environments in which examples of what urgently needs to be done in the fight to transform global food environments for people’s health are replicated and promoted, particularly those that are vulnerable [69]. It is essential to recognize the vital role of urban environments in achieving food sustainability in cities.

## 5. Conclusions

Unlike other studies [33] that also analyze patterns of change in the MAVM, this research addressed food environments through a spatial analysis that incorporated various scales and population characteristics to understand the problem from a perspective based on the solution of complex problems.

The proliferation of food swamps is a feature of food environments in the global south. Unlike food deserts in the global north, where solving physical access to healthier food suffices to regulate its effect on malnutrition, in food swamps in the global south, solutions must be geared towards solving physical access as well as the social preferences of the population for certain types of food.

The categorization and spatially explicit identification of food swamps, oases, and deserts can support interventions currently happening in the MVAM, such as the community dining rooms (CDRs) that serve nutritious meals at stable and subsidized prices (USD.50). To date, there are 488 CDRs serving 65,600 meals each day [70]. This research provided priority information regarding the most suitable sites to establish CDRs considering limited physical and economic access to food.

This methodological proposal will be extended to broader geographical areas, which would require applying these methods to the rest of the country’s urban contours. This challenge would make it possible to analyze the patterns of urban food environments in Mexico as a whole.

## Figures and Tables

**Table 1:** Food retail units.

Food Retail Units (DENUE Code)	Description
Supermarkets (462111)	Commercial establishments or self-service chains regarded as modern trade units. Characterized by having a broad, varied supply of fresh and packed food [39,40].
Convenience stores (462112)	Establishments with a multi-branch franchise format, with a broad geographical distribution, specializing in the sale of groceries, canned goods, and beverages [41,42].
Grocery stores: grocery store, corner shop, and general store (461110) Fresh fruit and vegetable store (461130) Red-meat store (461121) Poultry store (461122) Fish and seafood store (461123) Seeds, spices, and food grains store (461140)	Establishments in the form of corner shops and greengrocers, bulk seeds and the sale of meat, fish, and chicken. Considered traditional business units based on family self-employment [43]. Specializing in the sale of individual products in the case of stores selling vegetables, meat, chicken, and fish or a range of edible foods in the case of general stores [40].

Note: Supermarkets include public markets. The selected data correspond to raw material stores for food preparation. Fixed establishments dedicated to the sale of prepared food, such as restaurants and cafeterias, and itinerant markets are not considered, since they are regarded as a small sector compared to the variety of products in supermarkets, convenience stores, and local businesses. Source: [44,45] Delgado (1992); Teja & López (2013). Compiled by the authors.

**Table 2:** Urban food environments in the MAVM, 2010–2020.

Urban Food Environments (*n*) Number Of Blocks	2010			2020		
	Oases	Deserts	Swamps	Oases	Deserts	Swamps
	*n* = 11,149	*n* = 53,185	*n* = 2992	*n* = 35,822	*n* = 45,420	*n* = 7539
Central city (0)	87,701	202,796	151,846	137,222	31,854	272,738
First contour (1)	41,532	1,582,515	82,623	63,340	620,304	239,371
Second contour (2)	39,935	2,291,166	93,120	31,040	1,298,333	182,327
Third contour (3)	8781	2,302,102	57,770	17,359	1,566,886	212,731
Fourth contour (4)	180	879,690	5592	375	1,071,759	62,155
total	178,129	7,258,269	390,951	249,336	4,589,136	969,322

Note: (*n*) Number of blocks.
